# Health Care Utilization Before and After the “Muslim Ban” Executive Order Among People Born in Muslim-Majority Countries and Living in the US

**DOI:** 10.1001/jamanetworkopen.2021.18216

**Published:** 2021-07-30

**Authors:** Elizabeth A. Samuels, Lilla Orr, Elizabeth B. White, Altaf Saadi, Aasim I. Padela, Michael Westerhaus, Aarti D. Bhatt, Pooja Agrawal, Dennis Wang, Gregg Gonsalves

**Affiliations:** 1 Department of Emergency Medicine, Alpert Medical School of Brown University, Providence, Rhode Island; 2 Department of Political Science, Yale University, New Haven, Connecticut; 3 Department of Epidemiology of Microbial Diseases, Yale School of Public Health, New Haven, Connecticut; 4 Department of Neurology, Massachusetts General Hospital, Boston; 5 Department of Emergency Medicine, Medical College of Wisconsin, Milwaukee; 6 HealthPartners Center for International Health, St Paul, Minnesota; 7 Division of General Internal Medicine, University of Minnesota, Minneapolis; 8 Department of Emergency Medicine, Yale School of Medicine, New Haven, Connecticut; 9 Yale School of Medicine, New Haven, Connecticut

## Abstract

**Importance:**

The health effects of restrictive immigration and refugee policies targeting individuals from Muslim-majority countries are largely unknown.

**Objective:**

To analyze whether President Trump’s 2017 executive order 13769, “Protecting the Nation from Foreign Terrorist Entry into the United States” (known as the “Muslim ban” executive order) was associated with changes in health care utilization by people born in targeted nations living in the US.

**Design, Setting, and Participants:**

This retrospective cohort study included adult patients treated at Minneapolis-St. Paul HealthPartners primary care clinics or emergency departments (EDs) between January 1, 2016, and December 31, 2017. Patients were categorized as (1) born in Muslim ban–targeted nations, (2) born in Muslim-majority nations not listed in the executive order, or (3) non–Latinx and born in the US. Data were analyzed from October 1, 2019, to May 12, 2021.

**Exposures:**

Executive order 13769, “Protecting the Nation from Foreign Terrorist Entry into the United States.”

**Main Outcomes and Measures:**

Primary outcomes included the number of (1) primary care clinic visits, (2) missed primary care appointments, (3) primary care stress-responsive diagnoses, (4) ED visits, and (5) ED stress-responsive diagnoses. Visit trends were evaluated before and after the Muslim ban issuance using linear regression, and differences between the study groups after the executive order issuance were evaluated using difference-in-difference analyses.

**Results:**

A total of 252 594 patients were included in the analysis: 5667 in group 1 (3367 women [59.4%]; 5233 Black individuals [92.3%]), 1254 in group 2 (627 women [50%]; 391 White individuals [31.2%]), and 245 673 in group 3 (133 882 women [54.5%]; 203 342 White individuals [82.8%]). Group 1 was predominantly born in Somalia (5231 of 5667 [92.3%]) and insured by Medicare or Medicaid (4428 [78.1%]). Before the Muslim ban, primary care visits and stress-responsive diagnoses were increasing for individuals from Muslim-majority nations (groups 1 and 2). In the year after the ban, there were approximately 101 additional missed primary care appointments among people from Muslim-majority countries not named in the ban (point estimate [SE], 6.73 [2.90]; *P* = .02) and approximately 232 additional ED visits by individuals from Muslim ban–targeted nations (point estimate [SE], 3.41 [1.53]; *P* = .03).

**Conclusions and Relevance:**

Results of this cohort study suggest that after issuance of the Muslim ban executive order, missed primary care appointments and ED visits increased among people from Muslim-majority countries living in Minneapolis-St. Paul.

## Key Points

**Question:**

Was the 2017 “Muslim ban” executive order associated with changes in health care utilization by people born in Muslim-majority countries living in Minneapolis-St. Paul, Minnesota?

**Findings:**

This cohort study of 252 594 patients found that after the executive order was issued, there was an increase in missed primary care appointments and increased emergency department visits among people from Muslim-majority countries living in Minneapolis-St. Paul.

**Meaning:**

Changes in health care utilization among people from Muslim-majority countries after the Muslim ban may reflect changes in population health influenced by federal immigration policy.

## Teaser

This cohort study investigates the association between health care utilization and the so-called Muslim ban executive order for people born in Muslim-majority countries and living in Minneapolis-St. Paul, Minnesota.

## Introduction

The 2016 US presidential election was marked by anti-Muslim and anti-immigrant rhetoric, and the subsequent Trump administration introduced multiple restrictive immigration policies targeting individuals from Muslim-majority and Latin American countries.^1^ On January 27, 2017, President Trump issued executive order 13769, “Protecting the Nation from Foreign Terrorist Entry into the United States,”^2^ commonly referred to as the “Muslim ban.” The first iteration of the Muslim ban suspended the US Refugee Resettlement Program and prevented citizens from 7 Muslim-majority countries (Iraq, Syria, Iran, Libya, Somalia, Sudan, and Yemen) from traveling or immigrating to the US. The ban underwent multiple legal challenges but was upheld by the US Supreme Court in 2018.^3^ On January 20, 2021, President Biden repealed the Muslim ban by executive order.^4^

Policies like the Muslim ban exacerbate heightened levels of discrimination, hostility, and “othering” that US Muslims experience.^5^ Over the past 2 decades, there has been an increase in hate crimes^6^ and social hostility^7,8^ directed toward US Muslims—experiences that negatively affect health. Following the September 11 attacks, Arab Americans, including Muslim Arab Americans, experienced increased rates of anxiety, depression, and low birth weights.^5,9,10,11,12^ Islamophobia and restrictive entry policies have been associated with worse health outcomes among migrants, placing Muslim American immigrants and refugees at increased vulnerability as they are additionally targeted by immigration and refugee policy changes, above and beyond discrimination faced by US Muslims more broadly.^13,14,15^ Recent research has shown increased incidence of preterm birth among people from Muslim-majority countries after the Muslim ban was issued.^16^ However, it is largely unknown and can be difficult to measure how health and health care utilization in Muslim American immigrant and refugee communities changes in response to shifting sociopolitical climates due to several methodological challenges. National health and health care surveys, as well as administrative data sets, do not routinely capture religious affiliation, and naming and country-of-origin algorithms are not precise enough to distinguish people who are Muslim.^17,18^

To describe changes in health care utilization of people from Muslim-majority countries after enactment of the Muslim ban, we examined changes in primary care and emergency department (ED) utilization by people from Muslim ban–targeted nations living in the Minneapolis-St. Paul, Minnesota, metropolitan area, which is home to the largest Somali Muslim community in the US.^19^

## Methods

### Study Design

We conducted a retrospective, cohort study documenting trends in primary care and ED utilization, missed scheduled appointments, and stress-responsive conditions among individuals from Muslim ban–targeted nations living in Minneapolis-St. Paul 1 year before (January 1, 2016) to 1 year after (December 31, 2017) issuance of the ban. For outcomes with similar visit and diagnosis trends before the ban was issued, we used a difference-in-difference analysis to compare differences in utilization trends between people born in Muslim ban–targeted nations and non–Latinx US-born citizens. Supplementary analyses compared trends among people born in a Muslim-majority nation not listed in the Muslim ban to non–Latinx US-born citizens. Patient demographic, visit, and diagnosis data were extracted from the HealthPartners electronic health record (EHR) by a HealthPartners data analyst. HealthPartners and the institutional review board of Yale University approved this study and waived the need for patient informed consent. All records were deidentified and assigned a unique study identification number before secure filing from HealthPartners to the Yale analytic team. This study followed the Strengthening the Reporting of Observational Studies in Epidemiology (STROBE) reporting guideline.^20^

### Study Setting and Population

The Minneapolis-St. Paul metropolitan area has 3.63 million residents and the largest Somali Muslim population in the US. In 2017, approximately 252 000 area residents (59.3%) were White non-Latinx individuals, 81 900 (19.3%) were Black non-Latinx individuals, 26 800 (6.3%) were Asian non-Latinx individuals, and 40 900 (9.6%) were Latinx individuals.^21^ In 2016, there were approximately 17 889 people born in Muslim ban–targeted nations living in Minneapolis-St. Paul, of whom 15 808 (88.4%) were born in Somalia.^21^

We analyzed EHR data from HealthPartners, one of Minneapolis-St. Paul’s largest health care and insurance organizations, serving over 1.2 million patients at 55 primary care centers, 22 acute care centers, and 8 hospitals. Although religion is not recorded in the EHR, the HealthPartners EHR is unique in that it includes nation of origin information. This allowed us to characterize patients receiving care from January 1, 2016, to December 31, 2017, into 3 groups: (1) adults born in a country listed in the executive order (group 1) (Table 1; eTable 1 in the Supplement); (2) adults born in Muslim-majority nations not listed in the executive order (group 2) (Table 1, eTable 2 in the Supplement); and (3) US-born non–Latinx adults (Group 3) (Table 1). We excluded US-born Latinx patients (persons who identify as Latino, Latina, or Hispanic, or having lineage from Mexico or any nation in Central or South America) as they have been subject to distinct anti-immigrant rhetoric and policies that have important effects on their health and health care utilization.^22,23^

**Table 1.** Characteristics of HealthPartners Patients Seeking Care in a Primary Care Clinic or Emergency Department, January 2016 to December 2017^a^

Variable	No. (%)		
	Group 1: People born in a Muslim ban–targeted nation (n = 5667)^b^	Group 2: People born in a Muslim-majority nation not named in the Muslim ban (n = 1254)^c^	Group 3: US-born, non-Latinx (n = 245 673)
Race/ethnicity			
American Indian/Alaska Native	10 (0.2)	12 (1.0)	3207 (1.3)
Asian	45 (0.8)	307 (24.5)	5567 (2.3)
Black	5233 (92.3)	272 (21.7)	29 644 (12.1)
Native Hawaiian/Pacific Islander	5 (0.1)	11 (0.9)	660 (0.3)
White	155 (2.7)	391 (31.2)	203 342 (82.8)
Sex			
Female	3367 (59.4)	627 (50)	133 883 (54.5)
Male	2300 (40.6)	627 (50)	111 786 (45.5)
Age, y			
18-24	498 (8.8)	105 (8.4)	24 747 (10.1)
25-34	2076 (36.6)	283 (22.6)	49 897 (20.3)
35-44	1458 (25.7)	311 (24.8)	40 385 (16.4)
45-54	927 (16.4)	271 (21.6)	44 007 (17.9)
55-64	520 (9.2)	175 (14)	49 107 (20)
≥65	408 (7.2)	172 (13.7)	46 586 (19)
Insurance status			
Commercial	995 (17.6)	622 (49.6)	145 161 (59.1)
Medicare or Medicaid	4428 (78.1)	574 (45.8)	91 253 (37.1)

^a^ Missing or unknown data not included in table; sums may not add to 100%.

^b^ Iraq, Syria, Iran, Libya, Somalia, Sudan, and Yemen (eTable 1 in the Supplement).

^c^ Afghanistan, Albania, Algeria, Azerbaijan, Bahrain, Bangladesh, Bosnia-Herzegovina, Brunei, Burkina Faso, Chad, Cocos Islands, Djibouti, Egypt, Gambia, Guinea, Indonesia, Jordan, Kazakhstan, Kosovo, Kuwait, Kyrgyzstan, Lebanon, Malaysia, Maldives, Mali, Mauritania, Mayotte, Morocco, Niger, Oman, Pakistan, Palestine, Qatar, Saudi Arabia, Senegal, Sierra Leone, Tajikistan, The Comoros, Tunisia, Turkey, Turkmenistan, United Arab Emirates, Uzbekistan, Western Sahara (eTable 2 in the Supplement).

### Outcomes

We hypothesized that after issuance of the Muslim ban, we would observe increased health care visits for stress-responsive diagnoses, increased primary care missed appointments, and increased ED visits. Primary outcomes included the number of (1) primary care visits, (2) missed primary care appointments, (3) primary care stress-responsive diagnoses, (4) ED visits, and (5) ED stress-responsive diagnoses.

#### Primary Care Utilization

Primary care visits, missed appointments, and stress-responsive diagnoses were analyzed as counts per 1000 people. Kept and missed visit trends were examined overall, regardless of diagnosis, and stress-responsive diagnoses were analyzed separately. We identified stress-responsive diagnoses through literature review and opinion by study team members with expertise in Muslim, immigrant, and refugee health and who provide primary care to people from Muslim-majority countries living in Minneapolis-St.Paul.^9,23,24,25,26,27,28,29,30^ Diagnoses considered to be stress responsive were agreed upon by consensus (eTable 3 in the Supplement) and included 138 *International Statistical Classification of Diseases and Related Health Problems, Tenth Revision (ICD-10),* codes in 6 categories: mental health, sleep disorders, gastrointestinal symptoms, neurologic symptoms, food-related disorders, and pain syndromes.

#### Emergency Department Utilization

Overall ED visits and ED stress-responsive diagnoses were also analyzed as counts per 1000 people. As previously described, ED stress-responsive diagnoses were identified through literature review and expert opinion, were agreed upon by consensus, and included 27 *ICD-10* codes for acute coronary syndrome, assault, suicide attempt, and syncope (eTable 4 in the Supplement)^9,23,24,25,26,27,28,29,30,31,32^ as well as ambulatory sensitive conditions. Ambulatory sensitive conditions are conditions for which an ED visit or hospitalization is considered preventable through outpatient interventions and can be exacerbated by social stressors and inequalities.^31,33^ Ambulatory sensitive diagnoses included 21 *ICD-10* codes for angina, asthma, congestive heart failure, chronic obstructive pulmonary disease, diabetes complications, and hypertension (eTable 4 in the Supplement).^31,33^

### Statistical Analyses

We used local linear regression to characterize visit and diagnosis trends in 2016 and 2017 and fit separate models for all outcomes: primary care visits, missed primary care appointments, primary care stress-responsive diagnoses, ED visits, and ED stress-responsive diagnoses. After documenting changes over time, we identified outcomes for which trends appeared to be similar among non–Latinx US-born individuals and individuals from Muslim ban–targeted nations in the preintervention period by examining the interaction between study group and time (30-day periods) in linear regression models. For outcomes that followed approximately parallel trends before the ban was issued, we conducted difference-in-difference analyses.

The primary difference-in-difference analyses estimated the change in outcomes between pre- and post-ban periods among individuals from Muslim ban–targeted nations above and beyond the change observed among non–Latinx US-born individuals. For the difference-in-difference analyses, we fit the linear regression model described in equation 1:

*Y* = β_0_ + (β_1_Muslim ban targeted) + (β_2_Muslim ban targeted × post-Muslim ban) + (β_t_ period) + ε.

We compared the 360 days before and after the ban was issued, divided into 24 distinct, 30-day periods. Each outcome *Y* is a count per person per 30-day time period. The designations *Muslim ban targeted* and *Muslim ban targeted* × *post-Muslim ban* represent being from a Muslim ban–targeted nation and being from a Muslim ban–targeted nation after the ban was issued, respectively. For each model, we estimated the average difference in differences (β_2_) over increasing time intervals centered on the ban issuance date, beginning with 30 days before and after the ban was issued and increasing by 30-day increments to 360 days before and after issuance.

Demographic details were not included in the primary analysis because they do not vary over time. However, it is important to account for demographic details, because there are important differences between study groups that could influence health care utilization. In order to account for different characteristics between study groups and assess robustness, we compared changes in health care utilization by individuals from Muslim ban–targeted nations to 2 subgroups of non–Latinx US-born individuals demographically similar to individuals in group 1 (Muslim ban-targeted): a matched comparison group and a synthetic control. First, we matched on age, sex, race/ethnicity, and insurance type (Medicaid or Medicare, or commercial) to reweight members of group 3 (non–Latinx, US born) and identify a subset of group 3 with similar characteristics as group 1 (Muslim ban–targeted). We used the R package MatchIt, version 3.6.1, (R Core Team) to identify this reference group, then fit a weighted version of the model described in equation 1 (eTable 10 in the Supplement).^34^ Second, we used a generalized synthetic control method to reweight members of group 3 to produce a reference group with demographic characteristics and pre–Muslim ban outcomes more similar to those observed in group 1. Models were fit using the R package gsynth with parametric bootstrap standard errors.^35^ These robustness checks are reported in eTables 8-10 and in the eFigure in the Supplement. All *P* values were 2-sided, and *P* < .05 was considered significant. Data were analyzed from October 1, 2019, to May 12, 2021.

## Results

### Study Population Characteristics

From 2016 to 2017, 252 594 patients were included in this analysis: 5667 (2.2%) in group 1, 1254 (0.5%) in group 2, and 245 673 (97.3%) in group 3 (Table 1). People in group 1 were predominantly Black (n = 5233 [92.3%]), female (n = 3367 [59.4%]), aged 25 to 44 years (n = 3534 [62.3%]), and insured by Medicare or Medicaid (n = 4428 [78.1%]). People in group 2 were predominantly White (n = 391 [31.2%]), aged 25 to 54 years (n = 865 [69%]), and commercially insured (n = 622 [49.6%]). People in group 3 were predominantly White (n = 203 342 [82.8%]), female (n = 133 883 [54.5%]), aged older than 55 years (n = 95 693 [39.0%]), and commercially insured (n = 145 161 [59.1%]) (Table 1). The majority of people in group 1 were born in Somalia (n = 5231 [92.3%]) (eTable 1 in the Supplement). Approximately one-third of group 2 was from either Pakistan (n = 213 [17.0%]) or Egypt (n = 177 [14.1%]) (eTable 2 in the Supplement).

### Trends in Health Care Utilization and Diagnoses

**Figure 1.**
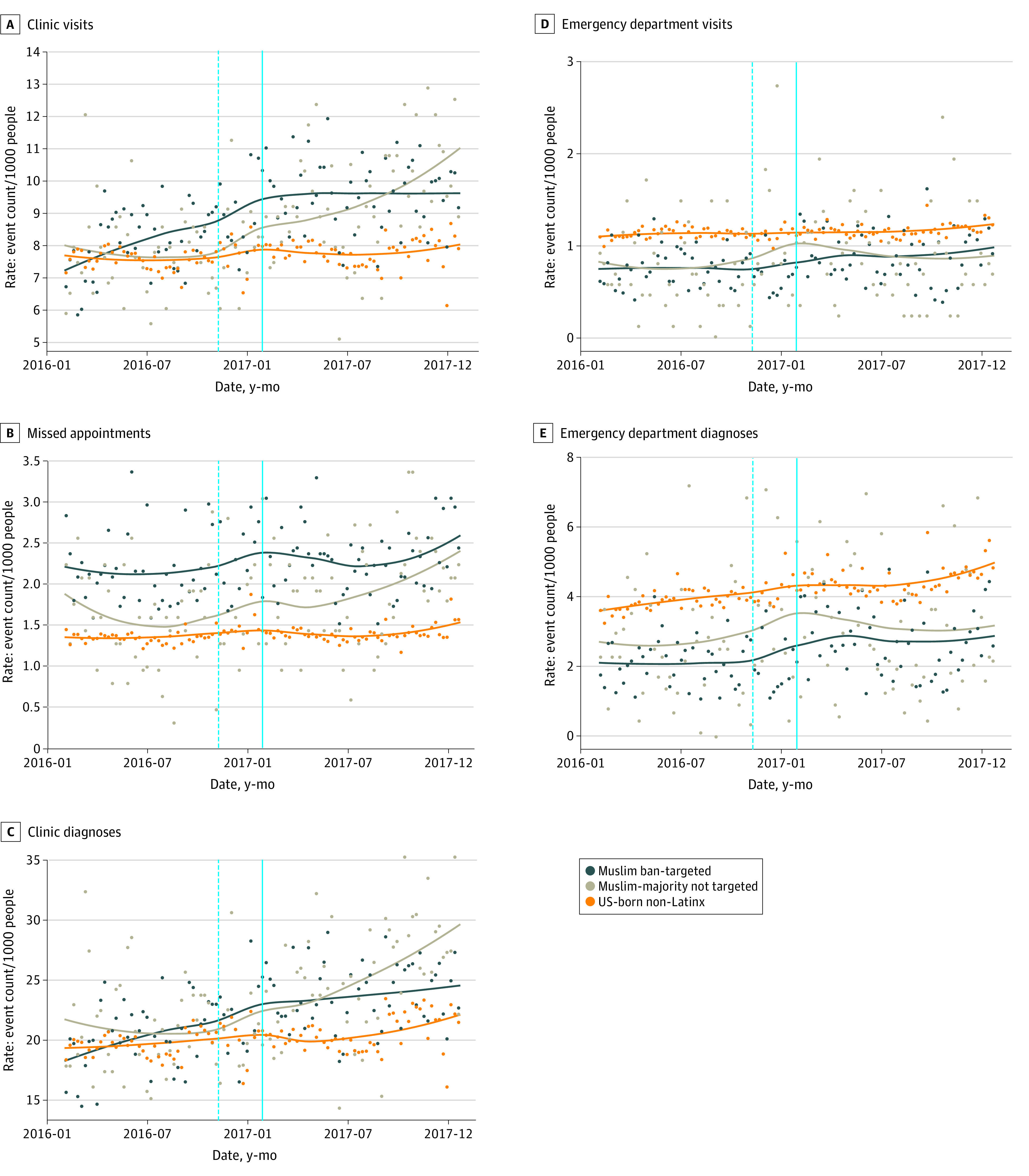
Time Trends for All Primary Outcomes Among Patients From Muslim Ban–Targeted Nations, Patients From Other Muslim-Majority Nations, and US-Born Non–Latinx Patients, January 2016 to December 2017 Points indicate weekly average counts per 1000 people in each group for (A) clinic visits, (B) missed clinic appointments, (C) clinic stress-responsive diagnoses, (D) emergency department (ED) visits, and (E) ED stress-responsive diagnoses. A LOESS regression line summarizing the time trend is included for each group, based on daily average counts per person. For all clinic outcomes, nonbusiness days are excluded from the analysis. The solid line indicates the Muslim ban issuance, and the dotted line indicates the 2016 election, for reference.

Similar trends between study groups were observed before Muslim ban issuance for missed primary care appointments, ED visits, and ED stress-responsive diagnoses. Figure 1 displays weekly average visit counts per 1000 people for each group, along with a local linear (LOESS) approximation of the time trend. The eFigure in the Supplement displays visit trends after demographic matching.

#### Primary Care Utilization

Mean rates of daily clinic visits and stress-responsive diagnoses were similar across all 3 groups in early 2016 (Figure 1A, 1C; eFigure in the Supplement). Although clinic visits and stress-responsive diagnoses remained fairly constant for US-born non-Latinx individuals (group 3), beginning in early 2016, they dramatically increased for individuals from Muslim-majority nations in groups 1 and 2 continuing after the presidential election on November 8, 2016 (Figure 1A, 1C, eTable 1 in the Supplement). This increase indicates that, before the Muslim ban, overall primary care visits and primary care stress-responsive diagnoses followed different trends across groups (eTables 5 and 6 in the Supplement). Missed scheduled appointments do appear to have followed approximately parallel trends before the ban was issued (Figure 1B; eTables 5 and 6 and eFigure in the Supplement), and so we additionally conducted a difference-in-differences analysis for this outcome.

#### Emergency Department Utilization

US-born non-Latinx individuals had higher baseline ED utilization and ED stress-responsive diagnoses; however, trends before the Muslim ban were fairly similar for all 3 groups (Figure 1D, 1E; eFigure in the Supplement). For groups 1 and 3, the rate of ED visits was mostly flat, whereas stress-responsive diagnoses slightly increased in 2016. During the 2016 election, the rate of ED visits and stress-responsive diagnoses increased for individuals from Muslim ban–targeted (group 1) and Muslim-majority nations (group 2) before leveling off at a higher utilization rate in mid to late 2017 (Figures 1A, 1C; eTables 5 and 6 and eFigure in the Supplement). Because ED visits and stress-responsive diagnoses appeared to have approximately parallel trends before the ban was issued, we conducted a difference-in-differences analysis for these outcomes.

#### Additional Change in Health Care Utilization and Diagnoses Following the Muslim Ban

**Figure 2.**
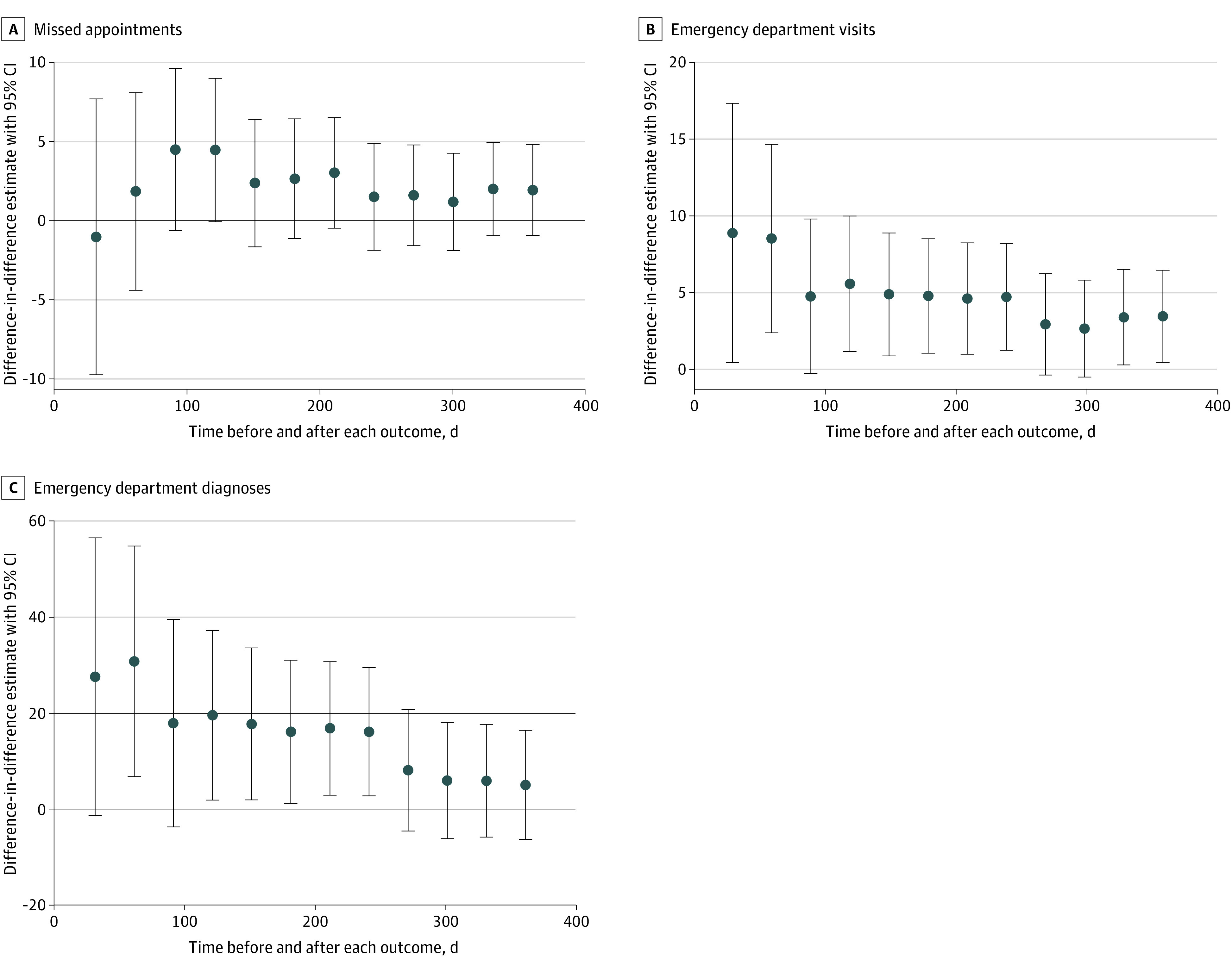
Difference-in-Difference Estimates for Missed Clinic Appointments, Emergency Department (ED) Visits, and ED Stress-Responsive Diagnoses, January 2016 to December 2017 Each point represents a difference-in-differences estimate in (A) missed appointments, (B) ED visits, or (C) stress-responsive ED diagnoses, with a 95% CI. The left-most points compare the difference in each outcome 30 days before to 30 days after the issuance of the Muslim ban for groups 1 and 3. Each additional point compares differences across a larger time period up to 360 days before and after the Muslim ban was issued.

For the 3 outcomes with similar trends before the Muslim ban across study groups (missed appointments, ED visits, and ED stress-responsive diagnoses), we used difference-in-difference analyses to estimate the magnitude of changes after the ban was issued within groups 1 and 2 above and beyond the change seen in group 3 (Table 2, Figure 2, eTable 7).

**Table 2.** Difference-in-Differences Estimates of Changes in Primary Care and Emergency Department Use After Muslim Ban Issuance

Outcome (mean per 1000 people)	Difference-in-differences model							
	Mean (SD)		Difference-in-differences estimate (SE)	*P* value	Matched difference-in-differences model estimate (SE)	*P* value		Generalized synthetic control model estimate (SE)
	US-born, non-Latinx	Muslim ban–targeted						
**Missed primary care appointments**								
Pre-Muslim ban	28.9 (197.0)	45.8 (240.1)	1.92 (1.47)	.19	2.99 (1.66)		.07	2.37 (1.17)
Post-Muslim ban	29.4 (119.4)	48.1 (250.7)						
First difference	0.4	2.4						
**ED visits**								
Pre-Muslim ban	31.9 (119.0)	21.2 (214.7)	3.41 (1.53)	.03	5.00 (2.34)		.03	4.41 (2.00)
Post-Muslim ban	32.9 (333.2)	25.6 (260.0)						
First difference	1.0	4.4						
**ED ambulatory sensitive and acute stress diagnoses**								
Pre-Muslim ban	119.0 (1269)	64.1 (727.7)	4.93 (5.79)	.39	−4.32 (9.09)		.63	19.3 (8.90)
Post-Muslim ban	133.3 (1428)	83.4 (956.8)						
First difference	14.4	19.3						

Abbreviation: ED, emergency department.

Rates of missed primary care appointments for group 1 did not change after the Muslim ban was issued to a significantly greater extent than rates changed in group 3 (difference-in-difference estimate [SE], 1.92 [1.47]) (Table 2, Figure 2A, eTables 7-9 in the Supplement). Similar estimates were also found when comparing group 1 to a demographically matched subset of group 3 and generalized synthetic controls (estimate [SE], 2.37 [1.17]) (Table 2).

We found a larger and statistically significant estimated additional increase in missed appointments for group 2 after the ban was issued (eTable 11 in the Supplement). The difference-in-difference point estimate for individuals from Muslim-majority nations not targeted in the Muslim ban was 6.73 (SE, 2.90; *P* = .02), suggesting that this group missed approximately 101 additional primary care appointments beyond what they would have been expected to miss if following the trend of non-Latinx US-born individuals.

##### Emergency Department Visits

In the year after the Muslim ban was issued, ED visits among individuals from Muslim ban–targeted nations significantly increased beyond the increase among US-born non-Latinx individuals (Table 2, Figure 2B). The difference-in-difference point estimate of 3.41 (SE, 1.53; *P* = .03) suggests that approximately 232 additional ED visits were made by individuals from Muslim ban–targeted nations in the 360 days after the Muslim ban was issued beyond what would have been estimated if ED utilization had followed a trend similar to that seen in group 3. This outcome was especially pronounced in the first 30 to 60 days after the ban was issued (Figure 2B). Estimates were similar with the matched and generalized synthetic control groups (difference-in-differences model estimate [SE], 5.00 [2.34] vs 4.41 [2.00]) (eTables 7-9 in the Supplement). For group 2, the estimated change was smaller and not statistically significant (eTable 11 in the Supplement).

##### Emergency Department Stress-Responsive Diagnoses

Primary difference-in-differences analysis found an increase in ED stress-responsive diagnoses after the ban was issued for individuals from Muslim ban–targeted nations relative to US-born non-Latinx individuals; however, this was not statistically significant (Table 2). The estimated change for Group 2 was also not significant (eTable 11 in the Supplement).

Following a similar pattern to ED visits, ED stress-responsive diagnoses increased more among individuals from Muslim ban–targeted nations than US-born non-Latinx individuals in the 30 to 60 days after the ban was issued (difference-in-differences estimate [95% CI], 27.44; 95% CI, −1.48 to 56.35 vs 30.64; 95% CI, 6.65 to 54.62) (Figure 2C).

## Discussion

Executive order 13769 was a major policy change restricting travel of immigrants and refugees from 7 Muslim-majority nations to the US. This cohort study found that after the Muslim ban was issued, there was an immediate and significant increase in ED visits among people from nations targeted by the ban. Primary care utilization and primary care visits for stress-responsive diagnoses were already increasing before the ban was issued, most notably after the 2016 presidential election. These findings may reflect elevated cumulative stress due to multiple restrictive policies and an increasingly hostile climate toward Muslim immigrants and refugees in the US and are consistent with other literature examining the health effects of restrictive entry policies in high-income countries.^6,7,8,13^

Estimated differences in ED utilization may seem small; however, they are average differences per 1000 people per 30-day period, and small per-person averages can result in substantial population health changes. All estimates may mask heterogeneity in health care utilization in a diverse population after the Muslim ban was issued, although the majority of people in group 1 were born in Somalia. Use of EHR data limits our ability to identify subgroups of people who may respond differently to stress. Individual-level factors, such as prior trauma, religion, acculturation, and a sense of belonging with one’s ethnic group, can influence coping.^17,29^ Factors not recorded in EHRs that may increase susceptibility to stress include prior negative interactions with the US immigration and/or refugee administration, changes in employment status, changes in comorbidities, and other immigration-related issues, such as awaiting family reunification and, for people who were refugees, prior time spent in a refugee camp (if any).^14,15,36,37^

Potential changes in utilization after the Muslim ban may have been attenuated by factors specific to Minneapolis-St. Paul and to the Somali American community there, which may not be present in other cities and states. Social capital and ethnic enclaves are important protective factors that may mitigate the harms from federal policy changes such as the Muslim ban. Social capital, or the ability to secure benefits through social networks and social structures, is strong among Somali Americans in Minneapolis-St. Paul compared with other US cities, including having elected representatives to city council and the US House of Representatives; such representation may attenuate the negative mental health effects of discrimination.^29,32,38,39,40^ Ethnic enclaves may protect immigrants from discrimination and related negative health effects. One study conducted after the 2016 presidential election found that residence in a Mexican enclave attenuated the risk of low birth weight for mothers of Mexican origin.^30^ A similar effect was demonstrated in a replication of a study by Lauderdale et al.^11,12^ The initial study demonstrated lower birth weights among Arab Americans after September 11, 2001, but this effect was not observed in Detroit, which has a large Arab American community.^12^

Research that aims to understand how immigration and refugee policy influences the health of Muslim immigrants and refugees is limited by the lack of available population-level data. Although increases in health care utilization may reflect increased community stress, this study did not directly measure stress or its association with health care utilization. It is important to consider that health care utilization may not be the most sensitive population-level outcome to use as a proxy measure for increased stress in immigrant communities.^22,31^ Although there are many differences in health care utilization by nation and regions of origin, overall, immigrants tend to have lower health care utilization compared with people born in the US, which may be influenced by multiple factors, including service accessibility, age, interpreter availability, acculturation, health-related beliefs, and comfort getting care within institutional medical establishments.^30,32^

### Limitations

There are several limitations to this study. First, evaluating population-level health changes among Muslim American immigrants and refugees after the Muslim ban was issued was challenging, as most EHR and health care data sources do not capture religious affiliation. As such, we used country of origin to estimate Muslim American immigrant and refugee health care utilization.^17,29,30^ Furthermore, we were studying a small population; as a result, only relatively large changes were easily measured. This may have resulted in an inability to detect small but important changes and precluded nuanced analyses of utilization trends.

Second, although we described trends in clinic utilization and stress-responsive diagnoses, we did not compare changes after the Muslim ban in these outcomes across groups, because trends diverged in the year before the ban was issued. Although we were examining changes in utilization around a distinct event, the Muslim ban was issued 7 days after President Trump’s inauguration, following a campaign characterized by anti-immigrant and anti-Muslim rhetoric, and it did not fully go into effect until June 2018. Therefore, estimated changes may have captured the cumulative effect of multiple events occurring around the time of the ban’s issuance and may not have reflected its full influence over time. Dedicated studies are needed to more fully evaluate the health effects of the 2016 election and the immigration and refugee policies of the Trump administration.

Third, Muslim American immigrants and refugees living in and around Minneapolis-St. Paul are not a homogenous group, and health care access and utilization may vary by countries of origin, ethnicities, and socioeconomic factors. Somali Americans, who are often racialized as Black, experience both anti-Black racism and anti-Muslim sentiment differently from Arab Muslim American immigrants and refugees racialized as White people. The compounded effects of Islamophobia, xenophobia, and anti-Black racism experienced by Somali Americans may have influenced changes in health and the health care utilization trends observed in this study. These circumstances are particularly relevant in Minneapolis-St. Paul, where in the wake of the murder of George Floyd, both the State of Minnesota and the Department of Justice have launched investigations into racist, discriminatory behavior in the Minneapolis police force. Further work is needed to better understand how anti-Black racism in Minneapolis-St. Paul, among police and in general, may influence health and decisions to utilize health care among Muslim American immigrants and refugees overall and Somali Americans in particular.

This study also did not account for second-generation immigrants or children of refugees who may experience social and familial stress due to the Muslim ban but would be included in the US-born comparison group, thereby reducing detected differences between study groups. Approximately one-quarter of US Muslims are born in the US with US-born parents (ie, nonimmigrant)^41^; our study did not capture any spillover effects on nonimmigrant US Muslim populations who may still be subject to discrimination following anti-Muslim rhetoric and policy changes. This study also did not examine spillover effects onto immigrant groups not from Muslim-majority countries, such as Latinx immigrants. Future studies are needed to fully evaluate these effects.

Because the majority of our study sample was composed of people from Somalia and the analysis was focused in 1 metropolitan area, the study group and location were not representative of all groups targeted by the Muslim ban, thereby limiting the generalizability of our findings. In other locations with smaller refugee and/or immigrant communities and fewer social supports, there may be larger negative outcomes associated with restrictive immigration and refugee policies.

## Conclusions

The results of this cohort study suggest that after issuance of the Muslim ban, ED visits increased among people from targeted Muslim-majority nations. Further investigations are needed to elucidate the health effects of restrictive immigration policies and cumulative social stress on Muslim American immigrants and refugees and identify protective factors that convey individual and population-level resilience to political social stressors.

## Notes

Supplement. **eTable 1.** Group 1 Country of Origin: People Born in a Muslim Ban Order Targeted Nation **eTable 2.** Group 2 Country of Origin: People Born in a Muslim-Majority Country Not Targeted by the Muslim Ban **eTable 3.** ICD-10 Codes Included in Analysis of Stress-Responsive Clinic Diagnoses **eTable 4.** Emergency Department Acute Stress Diagnoses and Ambulatory Sensitive Conditions **eTable 5.** Analysis of Linear Time Trends in Pre-Order Periods, Group 1 vs 3 (Unweighted) **eTable 6.** Analysis of Linear Time Trends in Pre-Order Periods, Group 1 vs 3 After Exact Matching on Demographics **eTable 7.** Difference-in-Differences Estimates of the Effect of the Muslim Ban Order on all Outcomes Among Patients From Order Targeted Nations (Group 1) **eTable 8.** Difference in Differences With Linear Time Trend **eTable 9.** Difference-in-Differences Estimates After Matching **eTable 10.** Characteristics of HealthPartners Patients Seeking Care in a Primary Care Clinic or Emergency Department Between January 2016 and December 2017, After Reweighting Through Matching on Demographics **eFigure.** Time Trends for all Primary Outcomes Among Patients From Muslim Ban-Targeted Nations and Demographically Matched US-Born non-Latin American Patients, January 2016 to December 2017 **eTable 11.** Difference in Differences Comparing Individuals Born in a Muslim-Majority Nation Not Named in the Muslim Ban Order and US-Born, Non-Latin American Individuals
