# Supplementary material for: Health Care Utilization Before and After the “Muslim Ban” Executive Order Among People Born in Muslim-Majority Countries and Living in the US
Source: JAMA Netw Open. 2021 Jul 30;4(7):e2118216. doi: 10.1001/jamanetworkopen.2021.18216 (PMC8325073; doi:10.1001/jamanetworkopen.2021.18216)
Supplement: Supplement. — eTable 1. Group 1 Country of Origin: People Born in a Muslim Ban Order Targeted Nation eTable 2. Group 2 Country of Origin: People Born in a Muslim-Majority Country Not Targeted by the Muslim Ban eTable 3. ICD-10 Codes Included in Analysis of Stress-Responsive Clinic Diagnoses eTable 4. Emergency Department Acute Stress Diagnoses and Ambulatory Sensitive Conditions eTable 5. Analysis of Linear Time Trends in Pre-Order Periods, Group 1 vs 3 (Unweighted) eTable 6. Analysis of Linear Time Trends in Pre-Order Periods, Group 1 vs 3 After Exact Matching on Demographics eTable 7. Difference-in-Differences Estimates of the Effect of the Muslim Ban Order on all Outcomes Among Patients From Order Targeted Nations (Group 1) eTable 8. Difference in Differences With Linear Time Trend eTable 9. Difference-in-Differences Estimates After Matching eTable 10. Characteristics of HealthPartners Patients Seeking Care in a Primary Care Clinic or Emergency Department Between January 2016 and December 2017, After Reweighting Through Matching on Demographics eFigure. Time Trends for all Primary Outcomes Among Patients From Muslim Ban-Targeted Nations and Demographically Matched US-Born non-Latin American Patients, January 2016 to December 2017 eTable 11. Difference in Differences Comparing Individuals Born in a Muslim-Majority Nation Not Named in the Muslim Ban Order and US-Born, Non-Latin American Individuals [file jamanetwopen-e2118216-s001.pdf]

Supplementary Online Content

Samuels EA, Orr L, White EB, et al. Health care utilization before and after the “Muslim ban” executive order among people born in Muslim-majority countries and living in the US. *JAMA Netw Open*. 2021;4(7):e2118216. doi:10.1001/jamanetworkopen.2021.18216

eTable 1. Group 1 Country of Origin: People Born in a Muslim Ban Order Targeted Nation

eTable 2. Group 2 Country of Origin: People Born in a Muslim-Majority Country Not Targeted by the Muslim Ban

eTable 3. ICD-10 Codes Included in Analysis of Stress-Responsive Clinic Diagnoses

eTable 4. Emergency Department Acute Stress Diagnoses and Ambulatory Sensitive Conditions

eTable 5. Analysis of Linear Time Trends in Pre-Order Periods, Group 1 vs 3 (Unweighted)

eTable 6. Analysis of Linear Time Trends in Pre-Order Periods, Group 1 vs 3 After Exact Matching on Demographics

eTable 7. Difference-in-Differences Estimates of the Effect of the Muslim Ban Order on all Outcomes Among Patients From Order Targeted Nations (Group 1)

eTable 8. Difference in Differences With Linear Time Trend

eTable 9. Difference-in-Differences Estimates After Matching

eTable 10. Characteristics of HealthPartners Patients Seeking Care in a Primary Care Clinic or Emergency Department Between January 2016 and December 2017, After Reweighting Through Matching on Demographics

eFigure. Time Trends for all Primary Outcomes Among Patients From Muslim Ban-Targeted Nations and Demographically Matched US-Born non-Latin American Patients, January 2016 to December 2017

eTable 11. Difference in Differences Comparing Individuals Born in a Muslim-Majority Nation Not Named in the Muslim Ban Order and US-Born, Non-Latin American Individuals

This supplementary material has been provided by the authors to give readers additional information about their work.

© 2021 Samuels EA et al. *JAMA Network Open.*

eTable 1. Group 1 Country of Origin: People Born in a Muslim Ban Order Targeted Nation (n=5,667)

Country	N (%)
Somalia	5231 (92.31)
Iraq	142 (2.51)
Sudan	130 (2.29)
Iran	109 (1.92)
Syria	29 (0.51)
Yemen	20 (0.35)
Libya	6 (0.11)

© 2021 Samuels EA et al. *JAMA Network Open.*

eTable 2. Group 1 Country of Origin: People Born in a Muslim-Majority Country Not Targeted by the Muslim Ban (n=1,254)

**Country**	**N (%)**
Pakistan	213 (16.99)
Egypt	177 (14.11)
Bosnia And Herzegovina	98 (7.81)
Sierra Leone	91 (7.26)
Afghanistan	84 (6.70)
United Arab Emirates	70 (5.58)
Bangladesh	58 (4.63)
Turkey	53 (4.23)
Guinea	49 (3.91)
Jordan	48 (3.83)
Lebanon	47 (3.75)
Kuwait	40 (3.19)
Morocco	37 (2.95)
Malaysia	36 (2.87)
Indonesia	33 (2.63)
Gambia	23 (1.83)
Djibouti	16 (1.28)
Algeria	14 (1.12)
Tunisia	11 (0.88)
Senegal	9 (0.72)
Uzbekistan	8 (0.64)
Bahrain	6 (0.48)
Azerbaijan	5 (0.40)
Kazakhstan	5 (0.40)
Albania	4 (0.32)
Burkina Faso	3 (0.24)
Kosovo	2 (0.16)
**Country**	**N (%)**
Kyrgyzstan	2 (0.16)
Mali	2 (0.16)
Niger	2 (0.16)
Qatar	2 (0.16)
Tajikistan	2 (0.16)
Brunei	1 (0.08)
Maldives	1 (0.08)
Oman	1 (0.08)
Turkmenistan	1 (0.08)

© 2021 Samuels EA et al. *JAMA Network Open.*

ICD-10 codes used in analysis of stress-responsive diagnoses

Prior to collecting or observing any data, research team members chose the following ICD-10 codes to include in analysis of stress-responsive diagnoses.

eTable 3. ICD-10 Codes Included in Analysis of Stress-Responsive Clinic Diagnoses

Category	Diagnoses	ICD-10 codes
Mental health	Panic disorder<br>GAD<br>Anxiety<br>New SI<br>Depression<br>Acute stress disorder<br>Adjustment disorder	F41.0 Panic disorder [episodic paroxysmal anxiety]<br>F41.1 Generalized anxiety disorder<br>F41.3 Other mixed anxiety disorders<br>F41.8 Other specified anxiety disorders<br>F41.9 Anxiety disorder, unspecified<br>F41 Other anxiety disorders (NB)<br>F51.5 Nightmare disorder<br>F32 Major depressive disorder, single episode (NB)<br>F32.0 Major depressive disorder, single episode, mild<br>F32.1 Major depressive disorder, single episode, moderate<br>F32.2 Major depressive disorder, single episode, severe without psychotic features<br>F32.3 Major depressive disorder, single episode, severe with psychotic features<br>F32.4 Major depressive disorder, single episode, in partial remission<br>F32.89 Other specified depressive episodes<br>F32.9 Major depressive disorder, single episode, unspecified<br>F33 Major depressive disorder, recurrent (NB)<br>F33.0 Major depressive disorder, recurrent, mild<br>F33.1 Major depressive disorder, recurrent, moderate<br>F33.2 Major depressive disorder, recurrent severe without psychotic features<br>F33.3 Major depressive disorder, recurrent, severe with psychotic symptoms<br>F33.40 Major depressive disorder, recurrent, in remission, unspecified<br>F33.41 Major depressive disorder, recurrent, in partial remission<br>F33.42 Major depressive disorder, recurrent, in full remission<br>F33.9 Major depressive disorder, recurrent, unspecified<br>F31.30 Bipolar disorder, current episode depressed, mild or moderate severity, unspecified<br>F31.9 Bipolar disorder, unspecified<br>F06.31 Mood disorder due to known physiological condition with depressive features<br>F43.0 (see below)<br>F43.1 Post-traumatic stress disorder (PTSD) (NB)<br>F43.10 Post-traumatic stress disorder, unspecified<br>F43.11 Post-traumatic stress disorder, acute<br>F43.12 Post-traumatic stress disorder, chronic<br>F43.0 Acute stress reaction<br>F43.8 Other reactions to severe stress<br>F43.9 Reaction to severe stress, unspecified<br>R45.7 State of emotional shock and stress, unspecified<br>R46.6 Undue concern and preoccupation with stressful events<br>Z63.7 Other stressful life events affecting family and household (NB)<br>Z63.79 Other stressful life events affecting family and household<br>Z73.3 Stress, not elsewhere classified<br>F43.2 Adjustment disorders (NB)<br>F43.20 Adjustment disorder, unspecified<br>F43.21 Adjustment disorder with depressed mood<br>F43.22 Adjustment disorder with anxiety<br>F43.23 Adjustment disorder with mixed anxiety and depressed mood<br>F43.24 Adjustment disorder with disturbance of conduct<br>F43.25 Adjustment disorder with mixed disturbance of emotions and conduct<br>F43.29 Adjustment disorder with other symptoms

© 2021 Samuels EA et al. *JAMA Network Open.*

Category	Diagnoses	ICD-10 codes
Sleep disorders	Insomnia	G47.0 Insomnia (NB)<br>G47.00 Insomnia, unspecified<br>G47.09 Other insomnia<br>F51.0 Insomnia not due to a substance or known physiological condition (NB)<br>F51.01 Primary insomnia<br>F51.02 Adjustment insomnia<br>F51.04 Psychophysiologic insomnia<br>F51.05 Insomnia due to other mental disorder<br>F51.09 Other insomnia not due to a substance or known physiological condition<br>Z72.820 Sleep deprivation<br>Z72.821 Inadequate sleep hygiene
Non-specific	Fatigue<br>Malaise	R53 Malaise and fatigue (NB)<br>R53.8 Other malaise and fatigue (NB)<br>R53.81 Other malaise<br>R53.83 Other fatigue
Gastrointestinal	GERD<br>PUD (H. pylori)<br>Abdominal pain (recurrent, NOS, flank pain, epigastric pain)<br>Irritable bowel	K21 Gastro-esophageal reflux disease<br>K21.0 Gastro-esophageal reflux disease with esophagitis<br>K21.9 Gastro-esophageal reflux disease without esophagitis<br>B96.81 Helicobacter pylori [H. pylori] as the cause of diseases classified elsewhere<br>K27 Peptic ulcer, site unspecified (NB)<br>K27.0 Acute peptic ulcer, site unspecified, with hemorrhage<br>K27.3 Acute peptic ulcer, site unspecified, without hemorrhage or perforation<br>K25 Gastric ulcer (NB)<br>K26 Duodenal ulcer (NB)<br>R10.10 Upper abdominal pain, unspecified<br>R10.11 Right upper quadrant pain<br>R10.12 Left upper quadrant pain<br>R10.13 Epigastric pain<br>R10.30 Lower abdominal pain, unspecified<br>R10.31 Right lower quadrant pain<br>R10.32 Left lower quadrant pain<br>R10.33 Periumbilical pain<br>R10.8 Other abdominal pain (NB)<br>R10.84 Generalized abdominal pain<br>R10.9 Unspecified abdominal pain<br>K58 Irritable bowel syndrome (NB)<br>K58.0 Irritable bowel syndrome with diarrhea<br>K58.1 Irritable bowel syndrome with constipation<br>K58.2 Mixed irritable bowel syndrome<br>K58.8 Other irritable bowel syndrome<br>K58.9 Irritable bowel syndrome without diarrhea<br>K59.1 Functional diarrhea
Neuro	Headache	G43 Migraine (NB)<br>G44 Other headache syndromes (NB)<br>G44.209 Tension Headache<br>G44.219 Tension Headache, episodic

© 2021 Samuels EA et al. *JAMA Network Open.*

Category	Diagnoses	ICD-10 codes
Food-related	Weight loss<br>Poor appetite	R63.5 Abnormal weight gain<br>R63.8 Other symptoms and signs concerning food and fluid intake<br>F50.89 Other specified eating disorder<br>R63.0 Anorexia<br>R63.4 Abnormal weight loss<br>R63.2 Polyphagia<br>O26.10 Low weight gain in pregnancy
Pain Syndromes	Chronic Pain<br>Back Pain	F45.41 Pain disorder exclusively related to psychological factors<br>F45.42 Pain disorder with related psychological factors<br>G89 Pain, not elsewhere classified (NB)<br>G89.2 Chronic pain, not elsewhere classified (NB)<br>G89.29 Other chronic pain<br>G89.4 Chronic pain syndrome<br>M79.601 Pain in right arm<br>M79.602 Pain in left arm<br>M79.603 Pain in arm, unspecified<br>M79.671 Pain in right foot<br>M79.672 Pain in left foot<br>R52 Pain, unspecified<br>M53.3 Sacrococcygeal disorders, not elsewhere classified<br>M54.2 Cervicalgia<br>M54.41 Lumbago with sciatica, right side<br>M54.42 Lumbago with sciatica, left side<br>M54.5 Low back pain<br>M54.6 Pain in thoracic spine<br>M54.8 Other dorsalgia (NB)<br>M54.89 Other dorsalgia<br>M54.9 Dorsalgia, unspecified

eTable 4. Emergency Department Acute Stress Diagnoses and Ambulatory Sensitive Conditions

Category	Diagnoses	ICD-10 codes
Stress-responsive diagnoses	Acute coronary syndrome<br>Assault<br>Suicide attempt<br>Syncope	I21.0-I21.A; I24.9<br>Y09., Y08.89x, X99.0xx-X92.9xx; Y01.xxx, Y97.xxx, Y38.3x1- Y38.3x3; T54.1x3-T54.3x3; X98.0xx-X98.9xx; X93.xxx- X95.9xx; Y03.8xx; Y02.0xx, Y00.xxx, Y04.0xx- Y04.2xx, Y04.8xx; Y08.01x- Y08.09x
Ambulatory sensitive conditions	Angina<br>Asthma<br>Congestive heart failure<br>COPD<br>Hypertension<br>Diabetes complications	I20, I24.0, I24.8–24.9<br>J45-46<br>Iii.0, I50, J81<br>J41-44, J47<br>I10, I11.9<br>E10.0–10.8, E11.0–11.8, E12.0–12.8, E13.0–13.8, E14.0–14.8

© 2021 Samuels EA et al. *JAMA Network Open.*

Analysis of pre-Order trends

In order to interpret the difference in differences presented in the manuscript as evidence of effects of the Muslim Ban, we would need to assume that in the absence of the executive order, the change in utilization and diagnoses would have been identical across groups. Although we cannot verify this assumption, we can observe whether or not differential trends appear before the executive order is issued. As the visualization in Figure 1 suggests, individuals from Muslim Ban targeted nations began to increase clinic utilization, relative to non-Latinx U.S.-born individuals, before the issuance of the executive order. Increases in stress-responsive diagnoses in the clinic are also observed before the executive order is issued. After restricting the comparison group through matching on patient demographics, these pre-Order trend differences are still present.

eTable 5. Analysis of Linear Time Trends in Pre-Order Periods, Group 1 vs 3 (Unweighted)

	Dependent Variable				
	Count of Visits or Diagnoses (Robust standard error)				
	Clinic Visits	Missed Appointments	Clinic Diagnoses	ED Visits	ED Diagnoses
Order Targeted	35.36 (4.79)	16.89 (2.00)	50.14 (12.14)	-11.90 (1.71)	-65.40 (6.01)
30 Day Period	-0.34 (0.09)	0.15 (0.03)	0.58 (0.25)	0. 03 (0. 05)	1.64 (0.22)
Order Targeted * 30 Day Period	3.13 (0.63)	0.006 (0.27)	5.01 (1.61)	-0.19 (0.23)	-1.62 (0.80)
Constant	158.30 (0.67)	29.86 (0.30)	419.89 (1.85)	32.07 (0.39)	129.62 (1.64)
Observations (N)	3016080	3016080	3016080	3016080	3016080
R^2	0.00003	0.00017	0.00001	0.00003	0.00006

eTable 6. Analysis of Linear Time Trends in Pre-Order Periods, Group 1 vs 3 After Exact Matching on Demographics

	Dependent Variable				
	Count of Visits or Diagnoses (Robust standard error)				
	Clinic Visits	Missed Appointments	Clinic Diagnoses	ED Visits	ED Diagnoses
Order Targeted	62.48 (5.21)	4.16 (2.26)	128.82 (13.34)	-64.17 (2.68)	-240.43 (10.00)
30 Day Period	-1.10 (0.31)	-0.19 (0.14)	-2.33 (0.81)	-0.23 (0.29)	3.89 (1.07)
Order Targeted * 30 Day Period	3.79 (0.69)	0.33 (0.31)	7.88 (1.78)	0.07 (0.36)	-3.85 (1.32)
Constant	129.86 (2.23)	42.35 (1.07)	339.48 (5.87)	84.40 (2.10)	304.82 (8.15)
Observations (N)	1303164	1303164	1303164	1303164	1303164
R^2	0.0003	0.00001	0.00019	0.00087	0.00078

© 2021 Samuels EA et al. *JAMA Network Open.*

Difference in differences estimates for all outcomes

Although we do not ascribe a causal interpretation to all difference in differences estimates, the full set of analyses originally planned are presented in Table S7. This is an expanded version of Table 2 in the main text.

eTable 7. Difference-in-Differences Estimates of the Effect of the Muslim Ban Order on all Outcomes Among Patients From Order Targeted Nations (Group 1)

**Outcome** (average per person)	**Difference in Differences Model**				**Matched Difference in Differences Model Estimate** (SE)	**Generalized Synthetic Control Model Estimate** (SE)
	Means (SD)	U.S.-born, non-Latinx	Order-Targeted	**Difference in Differences Estimate** (SE)		
Primary care visits	Pre-Order	160.5 (539.9)	175.5 (559.6)	**22.85**** (3.71)	**23.69**** (4.03)	**26.26**** (3.09)
	Post-Order	163.9 (552.6)	201.8 (620.8)			
	First Difference	**3.4**	**26.3**			
Missed primary care appointments	Pre-Order	28.9 (197.0)	45.8 (240.1)	**1.92** (1.47)	**2.99** (1.66)	**2.37** (1.17)
	Post-Order	29.4 (119.4)	48.1 (250.7)			
	First Difference	**0.4**	**2.4**			
Stress-responsive diagnoses in primary care	Pre-Order	416.1 (1476.5)	433.7 (1436.8)	**48.03**** (9.17)	**57.53**** (9.97)	**63.9**** (7.87)
	Post-Order	432.0 (1529.8)	497.6 (1574.0)			
	First Difference	**15.9**	**63.9**			
ED visits per person	Pre-Order	31.9 (119.0)	21.2 (214.7)	**3.41*** (1.53)	**5.00*** (2.34)	**4.41*** (2.00)
	Post-Order	32.9 (333.2)	25.6 (260.0)			
	First Difference	**1.0**	**4.4**			
ED ambulatory sensitive and acute stress diagnoses	Pre-Order	119.0 (1269)	64.1 (727.7)	**4.93** (5.79)	**-4.32** (9.09)	**19.3**** (8.90)
	Post-Order	133.3 (1428)	83.4 (956.8)			
	First Difference	**14.4**	**19.3**			

p < 0.05, p < 0.01***

Note: As in the main text, difference in differences estimates are additional increases in each outcome (per 1000 people per 30-day time period) from the year before to the year after the Muslim Ban was issued among individuals from targeted nations, beyond the increases observed in a reference group. Robust standard errors are included in parentheses for difference in difference estimates, clustered at the individual level, with and without demographic matching. Parametric bootstrap standard errors are included in parentheses for generalized synthetical control model estimates. The estimated sampling distributions do not necessarily follow an approximately normal distribution centered at the point estimate.

© 2021 Samuels EA et al. *JAMA Network Open.*

Robustness checks

To assess the robustness of the difference in differences analysis presented in the manuscript, we conducted two additional checks. First, we report estimates from a model which allows for a linear time trend as an alternative to period fixed effects. Second, we report regression coefficients and sample characteristics for the difference-in-differences models comparing the individuals from targeted nations to U.S.-born non-Latinx individuals with similar age, sex, race, and insurance to those observed for individuals from targeted nations. This reference group was selected using exact matching on all available demographics.

eTable 8. Difference in Differences With Linear Time Trend

	Dependent Variable				
	Count of Visits or Diagnoses (Robust standard error, clustered by individual)				
	Clinic Visits	Missed Appointments	Clinic Diagnoses	ED Visits	ED Diagnoses
Order Targeted	14.12<br>(3.49)	16.95<br>(1.25)	17.61<br>(8.30)	-10.66<br>(1.15)	-54.50<br>(4.20)
Order Targeted Post-Order	24.66<br>(3.70)	1.72<br>(1.47)	4.80<br>(9.16)	3.40<br>(1.53)	4.24<br>(5.76)
Period	0.12<br>(0.03)	0.05<br>(0.01)	1.22<br>(0.09)	0.08<br>(0.02)	1.16<br>(0.09)
Constant	162.20<br>(0.44)	29.14<br>(0.14)	434.03<br>(1.09)	32.39<br>(0.23)	126.15<br>(0.94)
Observations	6032160	6032160	6032160	6032160	6032160
Adjusted R2	0.00007	0.00018	0.00006	0.00002	0.00007

eTable 9. Difference-in-Differences Estimates After Matching

	Dependent Variable				
	Count of Visits or Diagnoses (Robust standard error, clustered by individual)				
	Clinic Visits	Missed Appointments	Clinic Diagnoses	ED Visits	ED Diagnoses
Order Targeted	37.86<br>(3.91)	2.04<br>(1.46)	77.57<br>(9.37)	-64.63<br>(1.95)	-215.38<br>(7.28)
Order Targeted Post-Order	23.69<br>(4.03)	2.99<br>(1.66)	57.53<br>(9.97)	5.00<br>(2.34)	-4.32<br>(9.09)
Constant	145.95<br>(3.73)	45.45<br>(1.66)	374.25<br>(9.78)	83.60<br>(3.20)	254.50<br>(10.44)
Period FE	Yes	Yes	Yes	Yes	Yes
Observations	2606328	2606328	2606328	2606328	2606328
Adjusted R2	0.00067	0.00009	0.00056	0.0008	0.00078

© 2021 Samuels EA et al. *JAMA Network Open.*

eTable 10. Characteristics of HealthPartners Patients Seeking Care in a Primary Care Clinic or Emergency Department Between January 2016 and December 2017, After Reweighting Through Matching on Demographics

	**Re-weighted after matching with race**	
	**Group 1**<br>**People born in an Order targeted nation**<br>(n = 5,654)<br>No. (%)	**Group 3**<br>**U.S.-born, non-Latinx**<br>(n = 102,943)<br>No. (%)
**Race**		
American Indian/Alaskan Native	10 (0.2)	182.1 (0.2)
Asian	44 (0.8)	801.1 (0.8)
Black	5,229 (92.5)	95,205 (92.5)
Native Hawaiian/Pacific Islander	5 (0.1)	91 (0.1)
White	155 (2.7)	2822.1 (2.7)
**Sex**		
Female	3,360 (59.4)	61,175.9 (59.4)
Male	2,294 (40.6)	111,786 (40.6)
**Age**		
18-24	498 (8.8)	9,227.6 (9)
25-34	2,076 (36.7)	37,448 (20.3)
35-44	1,458 (25.8)	26,587 (25.8)
45-54	921 (16.3)	16,432.5 (16)
55-64	518 (9.2)	9,371.7 (9.1)
≥65	403 (7.1)	7,392.8 (7.2)
**Insurance status**		
Commercial	995 (17.6)	18,116.1 (17.6)
Medicare or Medicaid	4,419 (78.2)	80,457.2 (78.2)

Note: As in Table 1, missing or unknown data not included in table; sums may not add to 100%. Re-weighted groups correspond to the comparison groups used when matching on all demographics.

© 2021 Samuels EA et al. *JAMA Network Open.*

eFigure. Time Trends for all Primary Outcomes Among Patients From Muslim Ban-Targeted Nations and Demographically Matched US-Born Non-Latin American Patients, January 2016 to December 2017

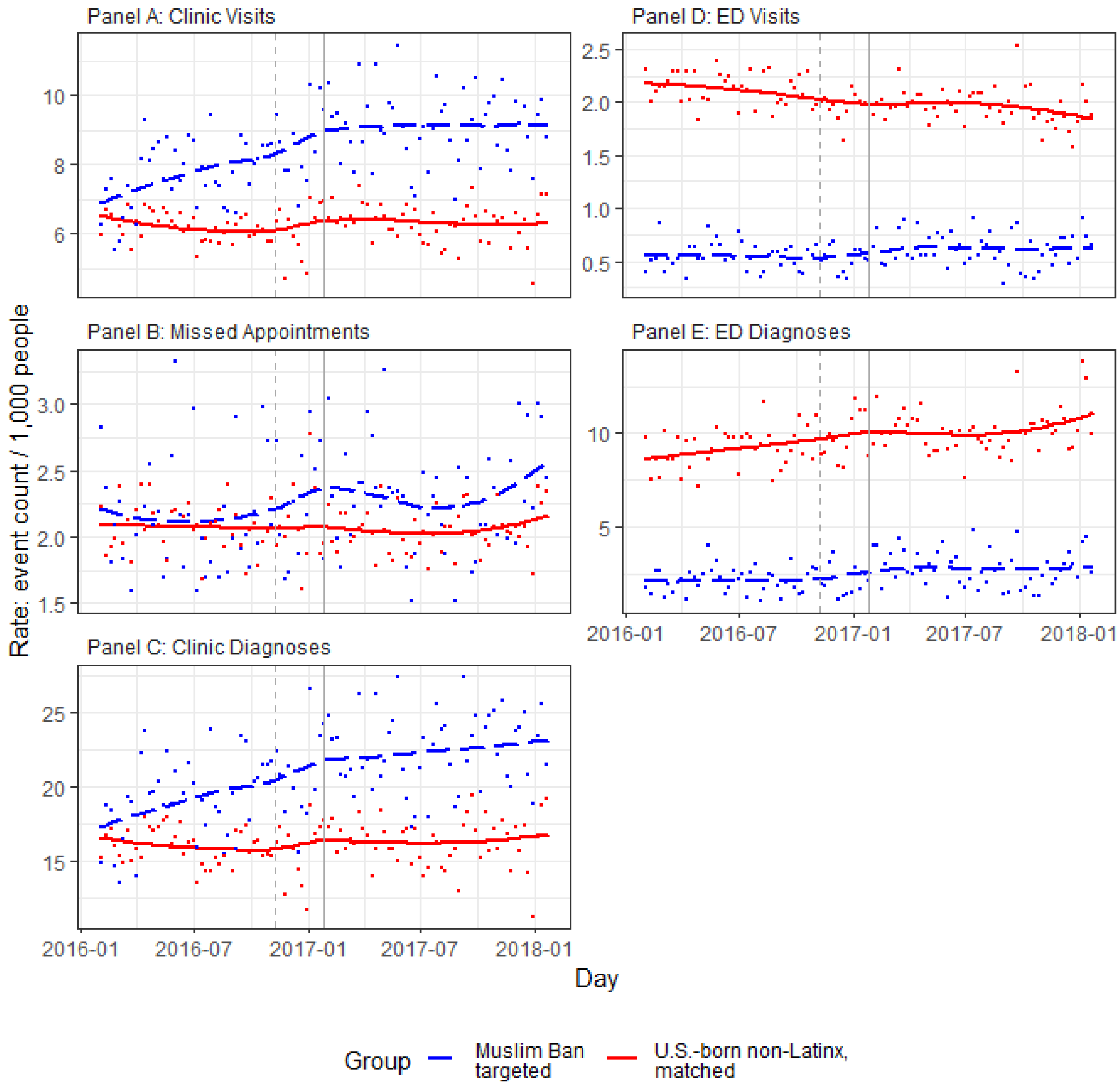

Points indicate weekly average counts per 1,000 people in each group after demographic matching for A) clinic visits, B) missed clinic appointments, C) clinic stress-responsive diagnoses, D) ED visits, and E) ED stress-responsive diagnoses. A LOESS regression line summarizing the time trend is included for each re-weighted group, based on daily average counts. For all clinic outcomes, panels A, B, and C, non-business days are excluded from the analysis. The solid line marks the Order issuance and the dotted line marks the 2016 election, for reference.

© 2021 Samuels EA et al. *JAMA Network Open.*

Differences following the Muslim Ban among individuals from Muslim-majority nations not targeted in the Order

Because we observed utilization and diagnosis trends only for 1,254 individuals from other Muslim-majority nations, we are not able to robustly compare the experiences of these individuals from Muslim-majority nations that were and were not named in the executive order. Nonetheless, we note that a difference-in-differences analysis comparing individuals born in a Muslim majority nation not named in the Muslim Ban and U.S.-born, non-Latinx individuals reveals qualitatively similar trends to those observed for individuals from targeted nations.

eTable 11. Difference in Differences Comparing Individuals Born in a Muslim-Majority Nation Not Named in the Muslim Ban Order and US-Born, Non-Latin American Individuals

	Dependent Variable				
	Count of Visits or Diagnoses (Robust standard error, clustered by individual)				
	Clinic Visits	Missed Appointments	Clinic Diagnoses	ED Visits	ED Diagnoses
Muslim majority	4.38 (7.36)	4.38 (2.21)	26.16 (18.92)	-9.10 (2.95)	-35.02 (14.97)
Muslim majority Post-Order	30.55 (7.72)	6.73 (2.90)	72.98 (20.74)	1.59 (3.30)	-1.89 (15.38)
Constant	169.00 (1.13)	30.13 (0.41)	431.18 (3.03)	30.51 (0.61)	107.71 (2.38)
Period FE	Yes	Yes	Yes	Yes	Yes
Observations (N)	5926248	5926248	5926248	5926248	5926248
R^2	0.00027	0.00007	0.00036	0.00002	0.00006

© 2021 Samuels EA et al. *JAMA Network Open.*
